# Genome-Wide Identification and Expression Analysis of NBS-Encoding Genes in *Malus x domestica* and Expansion of NBS Genes Family in Rosaceae

**DOI:** 10.1371/journal.pone.0107987

**Published:** 2014-09-18

**Authors:** Preeti Arya, Gulshan Kumar, Vishal Acharya, Anil K. Singh

**Affiliations:** Academy of Scientific & Innovative Research (AcSIR), New Dehli, India; Division of Biotechnology, CSIR-Institute of Himalayan Bioresource Technology, Palampur, Himachal Pradesh, India; Hellas, Greece

## Abstract

Nucleotide binding site leucine-rich repeats (NBS-LRR) disease resistance proteins play an important role in plant defense against pathogen attack. A number of recent studies have been carried out to identify and characterize NBS-LRR gene families in many important plant species. In this study, we identified NBS-LRR gene family comprising of 1015 NBS-LRRs using highly stringent computational methods. These NBS-LRRs were characterized on the basis of conserved protein motifs, gene duplication events, chromosomal locations, phylogenetic relationships and digital gene expression analysis. Surprisingly, equal distribution of Toll/interleukin-1 receptor (TIR) and coiled coil (CC) (1∶1) was detected in apple while the unequal distribution was reported in majority of all other known plant genome studies. Prediction of gene duplication events intriguingly revealed that not only tandem duplication but also segmental duplication may equally be responsible for the expansion of the apple NBS-LRR gene family. Gene expression profiling using expressed sequence tags database of apple and quantitative real-time PCR (qRT-PCR) revealed the expression of these genes in wide range of tissues and disease conditions, respectively. Taken together, this study will provide a blueprint for future efforts towards improvement of disease resistance in apple.

## Introduction

The battle between plants and pathogens is continued from ancient times. Thus, plants have evolved sophisticated mechanisms to identify and produce specific defense response against wide range of pathogens, including fungi, bacteria and insects [1]. The defense response in plants includes pattern recognition receptors (PRRs) and the cytoplasmic immune receptors. PRRs perceive conserved pattern associated with most of the pathogens, known as pathogen associated molecular patterns (PAMPs) whereas cytoplasmic immune receptors recognize factors secreted by pathogens directly or indirectly which in turn activates downstream signaling pathways leading to rapid local programmed cell death called hypersensitive response (HR). The defense response using cytoplasmic immune receptors is a well-known strategy characterized by specific interaction between disease resistance (*R*) genes of plants and corresponding avirulence (*avr*) genes of pathogen that results in disease resistance through hypersensitive response [2].

Numerous *R* genes have been cloned and characterized from different plant species during recent decades [3]. Most common *R* genes cloned to date, belong to the nucleotide binding sites and leucine rich repeats (NBS-LRR) family [4]. The NBS-LRR genes are the members of the STAND (Signal Transduction ATPase with Numerous Domains) family of NTPases and comprise the largest disease resistance gene family in plants [5]. These NBS-LRR genes encode proteins with amino-terminal variable domain, a central nucleotide binding site (NBS) and carboxy-terminal leucine rich repeats (LRR) domain [6]. The NBS domain was defined as a region of ∼300 amino acids containing several motifs arranged in specific order and is responsible for binding and hydrolysis of ATP and GTP during plant disease resistance whereas LRR motif is responsible for recognition of pathogen derived virulence factors in plant NBS-LRR proteins [6], [7], [8]. Based on the structural diversity in amino-terminal region, NBS-LRR family has been divided into two classes. The first class is termed as TIR-NBS-LRR (TNL) comprise of proteins containing the Toll/Interleukin-1 (TIR) receptor domain and the second is non-TIR-NBS-LRR (non-TNL) proteins that lack the TIR domain [1], [6]. In addition to their structural divergence, these two classes also differ in their downstream signaling pathways, thus possess functional divergence between them. Some members of non-TNLs class contain a predicted coiled coil (CC) structure in the amino-terminal region and thus are classified as CC-NBS-LRR (CNL) class. During phylogenetic analysis, it was observed that TNL and CNL class form distinct clades [4], [6], [8].

Genome wide investigation of NBS-LRR family in various plants species including rice [9], [10], *Arabidopsis thaliana* [8], papaya [11], *Vitis vinifera* [12], *Populus trichocarpa* [13], *Medicago truncatula* [14], *Brachypodium distachyon* [15] and *Solanum tuberosum* [16] have demonstrated the importance of these NBS-LRRs and also showed that they are highly duplicated and evolutionary diverse. It has been shown that NBS-LRR families contain high proportions of duplicated genes, and most of them are derived from tandem duplication events. This suggests that gene duplication events have played a major role in the expansion of this family [8], [10], [12], [17], [18].

Apple (*Malus domestica*) is one of the most economically important perennial fruit crops of the temperate zone. It belongs to the Rosaceae family that include many edible fruits such as cherries, plums, apricots peaches, pears, strawberries and raspberries, and economically important ornamental shrubs such as rose. It has been reported that the consumptions of apple by humans may reduce the risk of different kind of cancers [19]. Like all other plants, apple also faces an extensive damage in productivity because of various disease incidences. Among them fire blight (bacterial disease) and rust, black spot, Alternaria blotch and powdery mildew (fungal diseases) are the most common causes of production loss [20], [21], [22], [23]. Availability of apple genome [24] provides opportunities for identification, annotation and further analysis of disease resistance genes and to utilize functional genes to enhance disease resistance in apple.

In the present study, a complete set of NBS-LRR proteins (1015) was identified from the whole genome data set of apple. The family was characterized based on structural diversity among NBS-LRR proteins, annotations of functional domains using MEME, chromosomal location within the genome and identification of duplication events. The identified candidate proteins were further analyzed for comparative phylogeny between apple NBS-LRR proteins and functionally known NBS-LRR proteins of other related plant species. We have also performed digital expression analysis using an expressed-sequence tags (EST) database and quantitative real-time PCR (qRT-PCR) of selected genes under various disease conditions. This investigation will be helpful in selecting candidate disease resistance genes which would serve as a potential resource for improvement of disease resistance in apple.

## Methods

### Sequence Retrieval and identification of NBS-LRR family

Apple (*Malus x domestica* assembly v1.0) [24] protein sequences (63,517) downloaded from phytozome database (http://www.phytozome.net/apple.php) were used for prediction of NBS-LRR proteins [25]. The method used to identify NBS-LRR proteins in apple was identical to that of the previously described in case of other plants [11], [15], [16]. Meyers et al. [6], [8] defined NBS domain as a region ranging up to ∼300 amino acids that is composed of eight well-known characteristic motifs: P-loop (Kinase-1a), Kinase-2, RNBS-A, RNBS-B, RNBS-C, RNBS-D, GLPL and MHDV. Therefore, we performed thorough investigation of apple NBS-LRR protein sequences based on HMMER/Pfam results.

A set of candidate NBS-LRR proteins was identified from the complete set of predicted *M. domestica* proteins using a Hidden Markov Model (HMM) profile of the NBS (Pfam: PF00931) domain. Initially, the raw HMM profile of NBS downloaded from Pfam database v27.0 (http://pfam.sanger.ac.uk) [26] was searched against the apple protein sequences using module “hmmsearch” in the HMMER version V.3 with e-value<1e-04 [27]. We used two different strategies using HMMER results for further confirmation of NBS-LRR proteins. Firstly, all the protein sequences identified using “hmmsearch” was further analyzed using PfamScan to confirm the presence of NBS domain (ftp://ftp.sanger.ac.uk/pub/databases/Pfam/Tools/OldPfamScan.pfmascan.pl). Proteins with e-value larger than 1e-03 for NBS domain were excluded for further analysis.

In order to gain confidence in the first strategy, we followed another strategy for the construction of apple-specific NBS domain HMM profile to further assess NBS domain in the apple genome. This strategy was crucial to find the maximum number of candidate sequences. All the protein sequences searched using “hmmsearch” were initially restricted with an E-value cut off of less than 1e-60 and used as an input for ClustalW2 alignment [28]. The alignment file was then used for making apple-specific NBS HMM profile using module “hmmbuild”. Further, all predicted protein sequences of apple were searched using this apple-specific HMM profile and further confirmed for the presence of NBS domain using PfamScan results with E-value of less than 1e-04.

### Amino-terminal Analysis, classification and nomenclature of NBS-LRRs

The identified NBS-LRR proteins using both strategies were also searched against non-redundant (nr) database with BLASTP [29] and classified into regular and non-regular proteins [15]. The criterion for BLASTP hits used was >50% identities as well as 50% query coverage. For nomenclature prefix “Md” for *Malus domestica* was followed by NBS and numbered according to its distribution starting from regular to non-regular NBS-LRRs.

Both the TIR as well as LRR motifs in the regular and non-regular NBS-LRRs were identified in the Pfam results with keywords “TIR” and “LRR”. For identification of coil-coiled motif in these proteins we used COILS program (http://embnet.vital-it.ch/software/COILS_form.html) [30] with a threshold equal to 0.9. The results were then used to classify NBS-LRR family into seven classes.

### Analysis of Conserved motifs presents in NBS-LRRs

To investigate the structural motif diversity among the identified NBS-LRRs, the predicted NBS-LRR protein sequences were subjected to the motif analysis by MEME version 4.9.0 (Multiple Expectation Maximization for Motif Elicitation) [31]. MEME analysis was performed on the 808 regular NBS-LRRs from predicted candidate proteins. The criterion used for MEME analysis was (1) minimum width was 6; (2) maximum width was 20; (3) maximum number of motif was designed to identify 20 motifs; (4) the iterative cycles were set by default. As the sequences of non-regular NBS-LRRs are divergent or too short in their motif lengths, we have excluded non-regular NBS-LRRs from MEME analysis. MEME analyses were also done separately for each class of NBS-LRR (TNL, TN, CNL, CN, NL, N, TCNL or TCN) family.

### Mapping NBS-LRRs on chromosomes and gene duplication

Nucleotide sequences for all seventeen chromosomes were downloaded from NCBI (http://www.ncbi.nlm.nih.gov/genome/358) for mapping of NBS-LRR genes. The General Feature Format (GFF) file for apple genome annotation was downloaded from Phytozome database link (ftp://ftp.jgi-psf.org/pub/compgen/phytozome/v9.0/Mdomestica/annotation/Mdomestica_196_gene.gff3.gz) [25]. MapInspect software [32] was used for graphical representation of *M. domestica* NBS-LRR genes on chromosomes (http://www.plantbreeding.wur.nl/uk/software_mapinspect.html). A gene cluster is defined as the region in which two neighboring genes were less than 200 kb apart [33]. This definition of clustering was used to identify the organization of genes in a cluster on various chromosomes.

To search for potential duplicated NBS-LRRs in *Malus domestica*; MCScanX software (http://chibba.pgml.uga.edu/mcscan2/) was used [34]. All the protein sequences of apple were compared against themselves using all-vs-all BLASTP with parameters V = 10, B = 100, filter = seg, E-value<1e-10 and output format was set as tabular format (-m 8). The resulting blast hits were incorporated along with chromosome coordinates of all protein-coding genes as an input for MCScanX analysis. The resulting 63,517 hits were classified into various types of duplications including segmental, tandem, proximal, and dispersed under default criterion.

### Multiple Alignment and Phylogenetic Analysis

The identified NBS-LRR sequences of *M. domestica* along with well-known disease resistance genes in plant were aligned using ClustalW2 (Version 2.0.12) [28] with default parameters. The resulting alignment generated was used for phylogenetic analysis. Phylogenetic tree was constructed based on the Maximum Likelihood method with a GAMMA model and Blosum62 matrix using RAxML version 7.2.8 [35]. Topological robustness for each branch was assessed by bootstrap analysis with 100 replicates [36]. This analysis was carried out on 64-Intel Xeon core HP Proliant DL980G7 running Ubuntu 12.04.3 LTS operating system.

### Digital Gene Expression Analysis

*M. domestica* expression profile pattern were determined by searching the EST database of apple provided at the NCBI web site (http://www.ncbi.nih.nlm.gov/dbEST). Expression data of *M. domestica* was obtained using BLASTn searches on the EST database of *M. domestica* with an E-value of cut-off of 1e-10. Final expression pattern was obtained by removing those hits shorter than 200 base pairs as well as lesser than 95% identity.

### q-RT PCR Analysis of MdNBS genes

After the digital expression analysis, MdNBS genes which were found to be expressed only in shoot or leaves were selected. These MdNBS genes were aligned and phylogenetically clustered with known plant disease resistance genes using PhyML version 3.1 [37]. For qRT-PCR analysis, MdNBS genes were selected from the clades that contain any known plant resistance genes (Figure S1).

Pathogen infected leaf samples and insect-pest infected leaf sample were collected from orchard of local farmers of district Kullu (32°18′26.7′'N; 77°10′49.3′'E, Himachal Pradesh, India). Samples of leaf were collected and immediately frozen in liquid nitrogen and stored at −80°C for further use. Leaf samples infected with sap sucking insect-pest, virus and fungal pathogens were collected. Among fungal pathogen infected leaves, one was infected with powdery mildew and the two were infected with *Alternaria* with mild and severe symptoms, separately (Figure S2). The healthy leaf samples were used as control for relative expression analysis. The presence of fungal infection was confirmed through cloning and sequencing of ITS region using ITS1 and ITS4 primers. For the detection of virus, RT-PCR based identification method was followed as described by Kumar et al. [38]. The list of primer and their sequences are listed in Table S1. The total RNA was isolated as described by Muoki et al. [39], and cDNA was prepared using superscript-II (Invitrogen). The 1∶10 diluted cDNA was used for quantitative real time PCR (qRT-PCR) analysis of MdNBS genes. The primers were designed using Primer Express software version 3.0.1 (Applied Biosystems). The relative expression ratio of target genes as compared to respective control was calculated using REST 2009 software (Qiagen). Ribosomal protein L-2 (*RPL-2*) was used to normalize the qRT-PCR data.

## Results

### Identification and nomenclature of NBS-LRRs

Availability of *Malus x domestica* genome [24] made possible to identify and characterize the NBS-LRR family. We have followed two methods that were earlier proposed for the identification of NBS-LRRs. Initially, all the 63,517 predicted protein sequences downloaded from apple genome were scanned by “hmmsearch” module in HMMER using HMM profile of NB-ARC domain with E-value less than 1e-04, which resulted in identification of 1153 candidate proteins. These 1153 protein sequences were screened using PfamScan program for confirmation of NBS domain with significant E-value cut-off of 1e-04 which resulted in the identification of 1015 proteins.

We have also followed another strategy as proposed by Lozano et al. [16], using construction of apple-specific HMM (as described in Materials and Methods section), to gain confidence in the above described method. Both strategies confirmed the presence of 1015 NBS-LRRs in apple.

In the next step, we determined whether the identified 1015 hits belong to regular or non-regular proteins in accordance with the method followed in case of *Brachypodium* NBS-LRR family [15]. Through the comparison of nr database, we considered 808 hits as the regular NBS-LRR proteins which primarily showed ≥50% identity with the subject sequence of nr database, and the remaining hits (207) were defined as the non-regular NBS-LRR proteins. The motifs of NBS-LRR in these non-regular proteins were found to be either too divergent or too short in length. Thus, we restricted our analysis to a final set of 808 regular NBS-LRRs for phylogenetic analysis and motif elucidation. Apple NBS-LRRs were designated as MdNBS followed by number starting from 1 to 808 as regular and remaining as non-regular from 809 to 1015 (Table S2).

### Classification of NBS-LRR family

NBS-LRRs are characterized by presence of amino-terminal variable domain, a central nucleotide binding-site (NBS), and a carboxy-terminal leucine rich repeat (LRR). To further classify regular and non-regular NBS-LRRs, each of 1015 proteins were evaluated for amino- terminus; TIR domains and CC motif as well as carboxyl-terminus; LRRs. Thus, we classified all NBS-LRRs in seven classes: CC-NB-LRR (CNL), CC-NBS (CN), TIR-NBS-LRR (TNL) and TIR-NBS (TN), NBS-LRR (NL), NBS (N), and Mixed (TC) (Table S2). Mixed type NBS-LRRs include those which have both TIR and CC motif in amino-terminal region and further classified into two subclasses: TIR-CC-NBS-LRR (TCNL), and TIR-CC-NBS (TCN).

Out of 1015 NBS-LRR hits, 458 lack typical disease-resistance genes type domains without TIR domain or coiled-coil (CC) motif at the amino-terminal region and were distributed into NL (276) and N (182 have only NBS domain) classes. Remaining 557 MdNBSs were divided on the basis of amino-terminal region: with TIR domain (230), with coiled-coil (CC) motif (272) and mixture of both TIR and CC domain (TC) (55). MdNBSs having TIR domain were further classified into TNL (161) and TN (69) classes. The CC motif containing MdNBSs were also distributed into CNL (218) and CN (54) classes (Table 1).

**Table 1:** Number of NBS-LRRs reported in *Malus x domestica.*

Type of NBS genes	Class	Previous study*	Revised (Present Study)	Regular genes^a^
**TIR-NBS-LRR**	TNL	224	161	145
**TIR-NBS**	TN	20	69	68
**CC-NBS-LRR**	CNL	181	218	147
**CC-NBS**	CN	26	54	45
**NBS-LRR**	NL	394	276	199
**NBS**	N	104	182	153
**Mixed CC and TIR**	CT	43	55	
	CTNL	18	38	35
	CTN	5	17	16
**Total**		**992**	**1015**	**808**
**With LRR**		799	693	
**Without LRR**		193	322	

*Number of NBS-LRR genes in apple (*Malus x domestica* Borkh.) was taken from Velasco et al. [24].

a NBS-LRR genes considered for MEME and phylogenetic analyses in the present study.

We also found 27 MdNBSs with RPW8 (Resistance to Powdery Mildew 8) domain in the amino-terminal region. Out of these 27 MdNBSs, 12 were associated with coiled-coil domain and hence classified as CC_R_-NBS-LRRs [40] (Table S3).

### Analysis of Conserved motifs structures in regular NBS-LRRs

Among the disease resistance genes there is a known difference in signature motifs among the TNL and CNL classes [8]. Therefore, to investigate the structural divergence among 808 regular NBS-LRRs, we have considered all seven classes separately as a query for MEME. For further analysis, only those motifs were selected which have≥80% frequency of occurrence in a particular class. Previously, eight major motifs (P-loop, Kinase-2, RNBS-A, RNBS-B, RNBS-C, RNBS-D, GLPL and MHDV) were reported in the NBS region, most of them having different patterns depending on whether they are present in the TNL/TN or CNL/CN groups [6].

In the present study, the MEME result shows the presence of eight already known motifs in the MdNBSs confirming that the NBS domain is the most conserved region among all the domains encoded by disease resistance genes in plants. However, a difference in case of “MHDV” motif was found, where valine “V” is replaced by leucine “L” revealing “MHDL” motif in the majority of NBS resistance genes of apple. This “MHDV” motif is known in the majority of plant NBS-LRR families, which was reported in previous studies [6], [8]. Here, in apple MdNBSs, the MHDL motif was found to be present between the NBS domain and LRR motif which was also reported in the papaya NBS-LRR gene family [8], [11]. From MEME analysis of MdNBSs, the P-loop, Kinase-2, RNBS-B, GLPL and MHDL motifs showed high conservation levels, whereas RNBS-A, RNBS-C, and RNBS-D showed large variation in the conserved sequence between the different classes (Table 2). This study revealed that more than 95% of the regular NBS-LRRs contained at least five conserved regions in the NBS domain. Interestingly, in addition to known conserved motifs in NBS-LRRs, our analysis has identified some more conserved motifs with 80% frequency of occurrence in various classes of MdNBS family (Table 3).

**Table 2:** Well known conserved motifs specific to plant disease resistance genes found in apple.

Motif	P- loop	Kinase-2	RNBS-A	GLPL	RNBS-C	RNBS-B	RNBS-D	MHD-linker
**Class**								
**CN**	GMGGVGKTT	LLVLDDVW	FDLEKIQKE	CGGLPLA	WSLFEKMAF	GSKILVTTRS	QxLKPCFLYCSIFPK	-
**N**	GMGGLGKTT	LVVLDDVW	-	CGGLPLA	LELFSWHAF	GSRIIITTRD	FLDISCFF/CFLYCGLFPED	-
**TN**	GMGGIGKTTI	LLVLDDVDD	FLANVREx	AGGLPLA	ELFSWHAF	GSRIIITTRD	EKEIFLDIACFFKGM	-
**TCN**	GMGGLGKTT	LVIIDNVD	FLANVSETD	CGGLPLA	KLFSWHAF	GSRIIITTRD	FLDISCFF	-
**CNL**	GMGGVGKTT	LLVLDDVW	-	CGGLPLA	SEEESWSLFEKKAFx	GSKILLTTRD	LKRCFLYCSLFPKDY	MHDLVRD
**NL**	GMGGLGKTT	LLVLDDVW	-	CGGLPLA	LELFSxHAF	GSRIIITTRD	LKSCFLYCSIFPEDYEISxxRLIRL/FLDIACFF	MHDLLRE
**TNL**	GMGGIGKTT	LLVLDDVD	FLANVREx	AGGLPLA	LELFSWHAF	GSRIIITTRD	LDDNEKxIFLDIACFFKG	MHDLLRE
**TCNL**	GMGGLGKTT	LVIVDNVD	FLADVRET	CGGLPLA	LELFSWHAF	GSRIIITTRD	FLDISCFF	MHDLLRE

**Table 3:** Additional motifs (known/unknown) predicted by MEME analysis with frequency of occurrence > = 80% in the specific class of MdNBS genes.

Class (No. of MdNBS genes)	Motifs predictedthrough MEME analysis	Unknown/known	Occurrenceof motifs in MdNBSgenes (%)	Terminal(N: amino, C: carboxyl)	E-value	Width
**CC-NBS-LRR (157)**	IKELPESIGKLKHLRYLDLSG	Unknown	100	C	2.1e-1105	21
	xExEEKILPALKLSYDQLPSH	Unknown	84.07	Mid*	1.3e-672	21
	FPSLKxLxLRNCPKL	Unknown	88.53	C	9.9e-443	15
**NBS-LRR (189)**	TAIKELPxSIGxLxNLETLDL	Unknown	97.35	C	9.0e-941	21
	CxSLVELPxSIGKLINLRHLD	Unknown	91.00	C	4.3e-927	21
	LPSLEELxLxNCPNL	Unknown	86.77	C	2.8e-463	15
	LkxLPDSIGxLxSLRYLDLSG	Unknown	90.48	C	2.1e-556	21
**TIR-CC-NBS (16)**	VLPIFYHVDPSHVRKQNGSLAQAFQKHEE	Known^a^	100	N	8.7e-239	29
	SRISIIVFSKRYADSSWCLDELVKIMECR	Unknown	93.33	N	1.2e-224	29
	LVDLQEKLLSDILKQ	Unknown	93.33	Mid*	2.1e-039	15
	LERGEEIKEELFRAI	Unknown	86.67	N	2.2e-066	15
	EIKISSVDEGI	Unknown	86.67	Mid*	1.7e-014	11
**TIR-CC-NBS-LRR (35)**	EIKEELFRAIEESRISIIVFSKRYADSSWCLDELVKIMECR	Unknown	97.14	N	4.6e-844	41
	HVLPIFYHVDPSHVRKQDGSFAEAFQKHE	Known^a^	97.14	N	2.9e-605	29
	LGKLKILNLSHSxSLKKSPDFSGLPNLEEL	Unknown	91.43	C	1.0e-355	30
**TIR-NBS (68)**	SRISIIVFSKNYASSTWCLDELVKILECR	Unknown	77.94	N	1.8e-964	29
	VLPIFYDVDPSDVRKQKGSFA	Known^a^	92.65	N	4.7e-774	21
	GYEAELIEKIVEEIWxK	Unknown	98.46	N	1.2e-244	17
	LSVAKGLVGIDSRVEEISLLL	Unknown	92.65	N	3.0e-295	21
**TIR-NBS-LRR (145)**	TxGQIVLPIFYDVDPSDVRKQ	Known^a^	84.14	N	6.6e-1556	21
	GEDTRKGFTDHLYxALx	Unknown	84.14	N	7.6e-1200	17
	FSGLPNLEELILxGCKSLVEIHP	Unknown	93.80	C	4.6e-1088	23
	EKVxQWRDALTEAANLSGWDL	Unknown	96.55	N	3.0e-1141	21
	LexLxLNGCKRLQSIPDLP	Unknown	90.34	C	1.1e-758	19
	IPEDLGCLSSLExLDLSGNNFVSLPSSIG	Unknown	86.21	C	5.9e-1209	29
	FSTEAFSKMxNLRLL	Unknown	86.21	N	3.7e-648	15

a Motif known to be TIR specific Jupe et al. [33].

*Mid defines the region lies between amino and carboxyl terminal of NBS-encoding genes.

### Duplication pattern of NBS-LRR family in *Malus x domestica* genome

**Figure 1:**
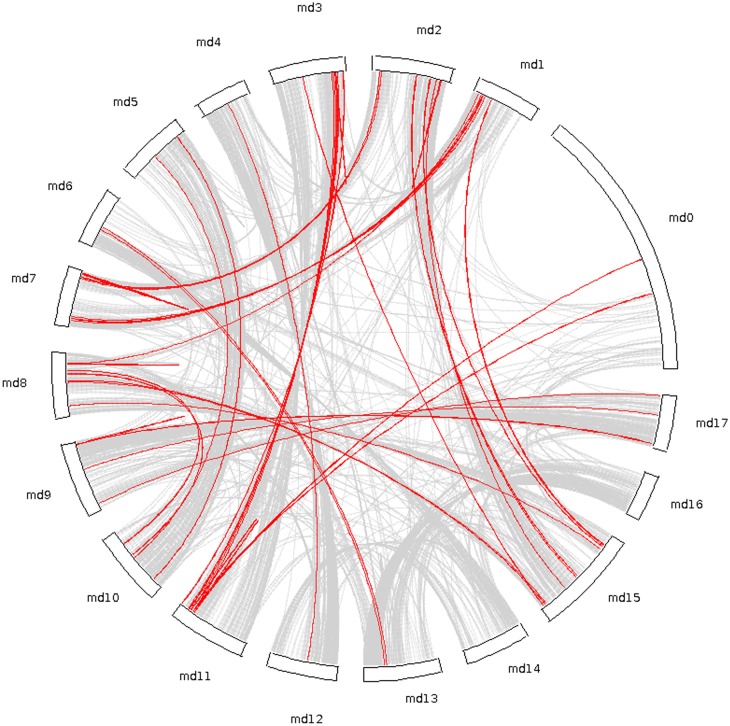
Collinear gene pairs for 1015 MdNBS genes on 17 apple chromosomes. Grey lines shows the collinear gene pairs in whole genome of apple whereas red lines show the collinear gene pairs for MdNBS genes.

Apple belongs to the Pyreae (Maleae), a subtribe in the family Rosaceae which shares duplication event with other eudicots [24]. During evolution, gene duplication has contributed to the expansion of gene families and establishment of new gene functions underlying the origins of evolutionary novelty [15], [41]. Sequencing and analysis of apple genome revealed that it has undergone a relatively recent (>50 million years ago) genome-wide duplication (GWD) event which results in the transition from nine ancestral chromosomes to 17 chromosomes [24]. The large size of MdNBS family suggests that it has evolved through a large number of duplication events in apple. Thus, in order to study the contribution of gene duplication events in expansion of MdNBS family, we analyzed whole genome duplication events in apple genome using MCScanX software. In whole genome of apple, we found 15,465 (24.35%) genes as segmentally duplicated and 10,812 (17.02%) as tandem duplicated genes. Among MdNBSs, 132 were found to be segmentally duplicated, which are located on duplicated segments on all chromosome except 14 and 16 (Figure 1 and Table S2). Maximum nineteen MdNBSs are located in duplicated segments on chromosome 11, followed by seventeen on chromosome 15, sixteen on chromosome 2, fifteen on chromosome 7, thirteen on chromosome 8, eleven on chromosome 9, nine on chromosome 1, four each on chromosome 17 and 10, three on chromosome 5 and two on chromosome 4. Duplicated segments on chromosome 6, 12 and 13 each contains one MdNBS gene. Remaining 7 segmentally duplicated MdNBSs were found to be unanchored. The McScanX analysis has also shown 254 MdNBS genes that were generated through tandem duplication which are present on all 17 chromosomes with the highest number (40) on chromosome 2.

### Chromosomal location and organization of apple NBS-LRRs

**Figure 2:**
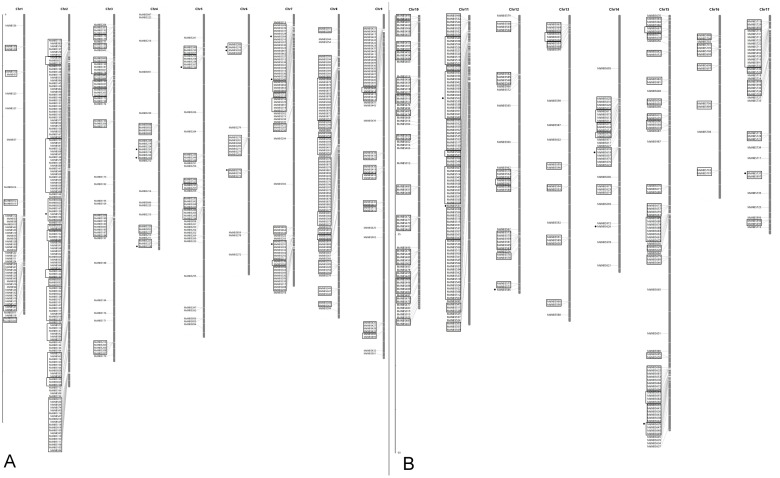
Physical location of MdNBS genes on chromosomes. (**A**) For chromosome (chr) 1, 2, 3, 4, 5, 6, 7, 8, and 9; (**B**) For chromosome (chr) 10, 11, 12, 13, 14, 15, 16, and 17. The number at the top of bar indicates the chromosome number. The scale used for plotting MdNBS genes is in megabases (Mb). The MdNBS genes cluster together were shown in black rectangular boxes. Those MdNBS genes expressed in qRT-PCR analysis were represented using a black solid circle.

The chromosomal position for all 1015 MdNBSs were identified by deploying the information as described in Mdomestica_196_gene.gff3 and graphically portrayed using MapInspect tool. The present study mapped 928 MdNBS (91.43%) genes which were further used to determine their chromosomal distribution. All the anchored MdNBS genes were found to be distributed on all the 17 apple chromosomes. The number of MdNBS genes located on chromosome 1 to 17 was 49, 145, 53, 29, 43, 18, 65, 88, 51, 69, 100, 33, 23, 32, 77, 14, and 39, respectively. The chromosome 16 has least number (14, 1.5%) of genes whereas chromosome 2 has highest number (145, 15.6%) of MdNBS genes. The clustering of genes showed that 751 (80.9%) of MdNBS genes were present in 159 clusters. The higher numbers of clusters were present on chromosome 2, 8, 10, 11, and 15 with clusters 15, 14, 15, 12, and 18, respectively. The single largest cluster comprised of 26 MdNBS genes was found to be located on chromosome 2. Approximately 24.6% (228 out of 928) of MdNBS genes clustered on chromosome 2 and 11 (Figure 2A and 2B).

**Figure 3:**
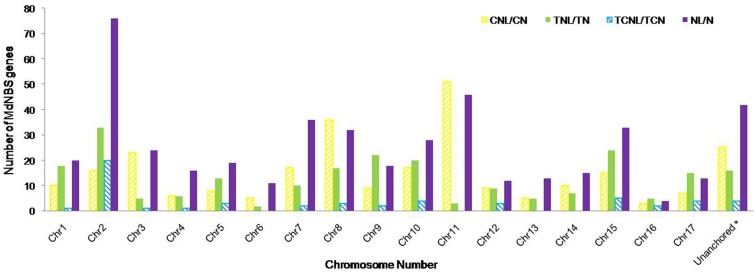
The distribution of NBS-LRRs (TNL/TN, CNL/CN, TCNL/TCN and NL/N) within the apple genome. The length of the bar shows the number of MdNBSs belongs to specific class located on each chromosome. *MdNBS genes not positioned on any of 17 chromosomes.

Furthermore, it was observed that chromosomal distribution of various classes of MdNBS genes (TNL/TN, CNL/CN, TCNL/TCN and NL/N) show slight bias, as shown in Figure 3. The TNL/TN class genes were present mainly on chromosome number 2, 9, 10 and 15; CNL/CN on chromosome 3, 8 and 11; TCNL/TCN on chromosome 2; whereas NL/N class genes were mainly present on chromosome 2 and 11. Chromosome 2 shows the highest number of MdNBS having TIR domain: TNL/TN (33) and TCNL/TCN (20). Chromosome 11 has highest number of CNL/CN type (51) and very less number of TNL/TN (3) MdNBS genes.

### Phylogenetic Analysis of NBS-LRRs

Phylogenetic relationships and evolutionary history in NBS-LRR family were inferred by constructing a phylogenetic tree using full length protein sequences of regular MdNBS and well-known disease resistance genes from different plant species *viz*, *Hordeum vulgare, Solanum lycopersicum, Solanum pimpinellifolium, Nicotiana tabacum, Oryza sativa, Arabidopsis thaliana,* and *Triticum aestivum* (Table S4). These 808 regular MdNBSs and 71 well-characterized resistance genes were aligned and a phylogenetic tree was generated.

**Figure 4:**
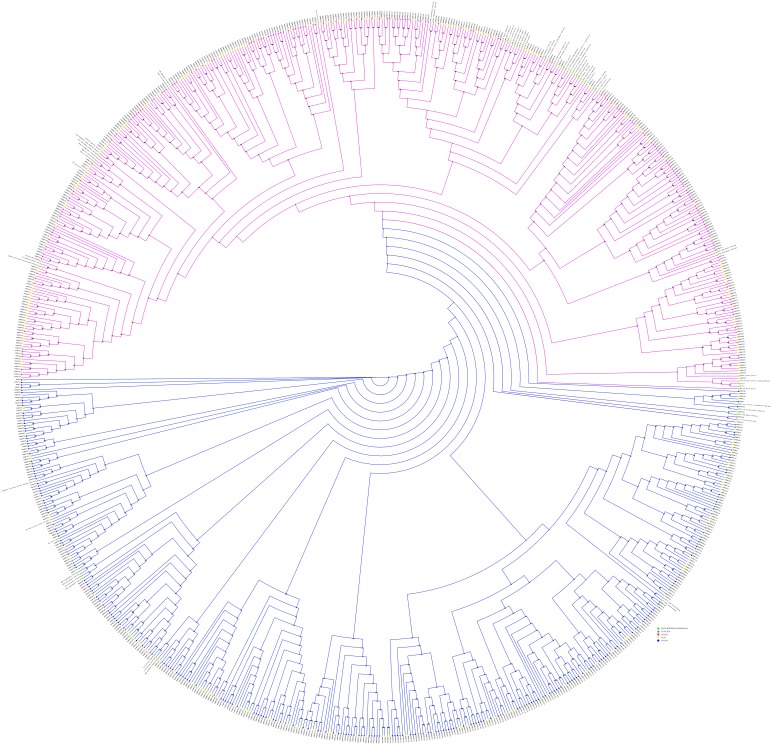
Phylogenetic tree of apple NBS-LRRs with the known plant disease resistance genes. TNL/TN types are shown as solid dark blue circles. CNL/CN types are shown as solid pink circles. Mixed types (TCN/TCNL) are shown as solid grey circles. NL/N types are shown as solid yellow circles. Solid green circles are used to represent already well-known plant disease resistance genes.

Phylogenetic reconstruction of NBS-LRRs (Figure 4) in apple shows a clear demarcation between TNL/TN and CNL/CN groups [6]. The members of NL/N class were unevenly dispersed throughout the phylogenetic tree which shows that NBS-LRRs have diverse origin [13]. NL/N can be further grouped as NL_CC_/N_C_ and NL_TIR_/N_TIR_ on the basis of presence in their respective clade. Interestingly, from the phylogenetic tree it can be inferred that TNL/TN type genes were evolved earlier than the CNL/CN type genes which has been hypothesized in earlier studies related to NBS-LRR family [42].

It was observed from the phylogenetic tree that TIR containing known plant disease resistance genes (L6, rust M, P2-A, RPS4, RPP1, RPP5 and RPP4) were grouped with TNL clade. Similarly coiled coil motif containing disease resistance genes (RPM1, RPW8, PizT, MLA1, MLA12, MLA6, MLA10 and Pi36) were found within CNL/CN clade. This comparative analysis may help in characterization of uncharacterized MdNBS family of apple.

### Digital expression patterns for MdNBS genes

The availability of *M. domestica* EST database makes possible to study the digital gene expression of MdNBS genes. On the basis of tissue and organ types, we assign the MdNBS genes to five different groups (shoot, root, leaf, phloem and xylem; Table S5) according to the gene expression patterns generated through digital gene expression analysis. It was observed that 361 out of 1015 MdNBS were supported by expression evidence after analysis and integration of gene expression data. The number of MdNBS genes was 56, 48, 82, 61, and 18 that expressed in shoot, root, leaves, phloem and Xylem, respectively. It was observed that 53 MdNBS genes were expressed in multiple tissues.

### Expression analysis of MdNBS genes using quantitative Real time PCR

**Figure 5:**
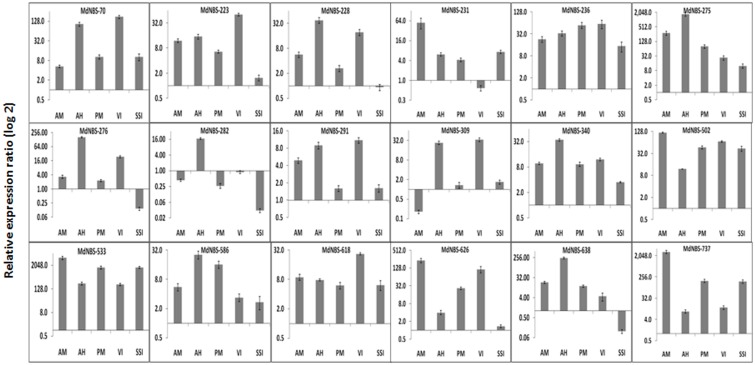
Relative transcript level of 18 MdNBSs obtained from quantitative real time PCR analysis in different pathogen infected leaf samples. The expression level was calculated relative to the healthy leaf sample. AM: mild infection of *Alternaria*, AH: heavy infection of *Alternaria,* PM: powdery mildew, VI: viral infect, SSI: sap sucking insect. Ribosomal protein L-2 gene was used as internal reference gene for data normalization.

From digital expression (in leaf and shoot tissues) and phylogenetic analysis with known disease resistance genes (Figure S3), a total of 26 MdNBS genes were selected and their expression profiling in naturally infected leaf samples was studied. Out of these 26, 18 MdNBS genes were found to be expressed, while expression of rest of the 8 genes was not observed in any of the conditions, studied. Symptomatic pathogen infected samples of Royal Delicious apple variety were collected from the apple orchard located at Kullu, Himachal Pradesh. The identification of fungal pathogens using ITS region sequencing confirmed the *Alternaria* (Alternaria) and *Podosphaera leucotricha* (Powdery mildew) pathogens. The sequencing of PCR amplified product of viral coat protein-coding region reveal the infection of *Apple Mosaic Virus*. The sap sucking insect was identified as *Rhopalosiphum insertum.* Interestingly, irrespective of the pathogen/insect pest infection, the expression of MdNBS70, MdNBS236, MdNBS275, MdNBS340, MdNBS502, MdNBS533, MdNBS586, MdNBS618 and MdNBS737 was found to be up-regulated in all the infected samples as compared to healthy ones. Similarly, expression of MdNBS228, MdNBS276, MdNBS626 and MdNBS638 was up-regulated in all the samples, except in the sample with sap sucking insect. In case of leaf samples with heavy *Alternaria* infection, expression of all the 18 MdNBS genes was up-regulated, while in case of mild *Alternaria* infection, expression of MdNBS282 and MdNBS309 was found to be down-regulated while remaining 16 MdNBS genes were up-regulated. Similarly, expression of all the 18 MdNBS genes was found to be up-regulated under powdery mildew infection except, MdNBS282 which was down-regulated and MdNBS291 and MdNBS309, which were unaltered. Moreover, virus infected leaf sample was shown to exhibit significant up-regulation of all the 18 MdNBS genes, except MdNBS231 and MdNBS282 whose expression was found to be down-regulated and unaltered, respectively. In case of sap sucking insect, the expression of three MdNBS *viz:* MdNBS276, MdNBS282, MdNBS638 was found to be down-regulated, while the remaining genes were either up-regulated or unaltered (Figure 5).

## Discussion

Apple (*Malus x domestica*) is one of the economically important fruit crops that is widely cultivated throughout the temperate zone of the world. Apple trees are susceptible to a number of diseases including fungal, bacterial and insect pests. To reduce the crop loss due to these diseases, understanding and improvement of disease resistance is crucial. With the availability of apple genome, it is possible to carry out genomic studies on NBS-LRR genes that confer resistance to rapidly evolving pathogens.

The NBS-LRR disease resistance genes have been studied extensively in various plant genomes such as *Arabidopsis* [8], *Solanum tuberosum* [16], [33], *Brachypodium distachyon* [15], *Glycine max* [43], *Oryza sativa* [9] and *Zea mays* [44]. In the present study, we found 1015 NBS-LRR hits using the apple proteome by iterative computational methods, whereas only 992 NBS-LRRs were reported in the previous study [24]. Perazzolli et al. [45] further identified 868 out of 992 NBS-LRRs as resistance gene analogs (RGAs) and remaining were considered as putative ones. The genes predicted by the present and previous studies are listed in Table S6.

The analysis described by Velasco et al. [24] was based on InterPro and Panther searches, which is most prominent web server for functional analysis of proteins by classifying them into protein families and domain. Perazzolli et al. [45] initially used HMMER and BLASTN search for identifying candidate genes by sharing significant protein similarity with known plant RGAs in *A. thaliana, P. trichocarpa,* and *V. vinifera.* While, we used two different methods, based on HMM and Pfam search and construction of apple-specific NBS hidden Markov model, both of which confirm the similar number of NBS-LRR proteins (1015) in apple. The number of NBS-LRRs which accounts for approximately 1.6% of all the genes which is in the expected lines with the all available plant sequenced genomes so far.

To determine the regular NBS-LRRs based on the presence or absence and short or large sequence length of motifs, we followed the strategy of excluding those NBS-LRRs which have <50% identity to the public available nr database that results in 808 regular NBS-LRRs. The genes encoding NBS-LRR proteins were classified into two broad groups (TNL and CNL) based on the amino-terminal region. There has been a known difference between these two groups on the basis of motifs identified by MEME. Our analysis supported the existence of distinction among the TNL/CNL groups. NBS-LRRs in apple genome constitute a total number of 230 TIR and 272 Coiled-coil domains in the amino-terminal region, making a ratio of approximately 1∶1 (CNL: TNL). While, a ratio of 1∶2 (CNL: TNL) has been reported in the Brassicaeae family including *A. thaliana, A. lyrata* and *Brassica rapa* [8], [46], [47] and a ratio of 4∶1 (CNL: TNL) observed both in potato [33] and grapevine genome [12]. The equal distribution of CNLs and TNLs in apple genome may suggest that both groups have an equal contribution in response to the pathogen attack.

Further, two groups (CNL and TNL) were subdivided into seven classes. The similar classes were also identified by Velasco et al. [24], however, a slightly higher number of CNL/CN and TN groups and a slightly lower number of TNL and NL groups were observed in the present study. In addition, we also found slight higher number of hits in mixed group (both CC and TIR present in the amino-terminal region) (Table 1).

We also considered MEME output which has >80% frequency of occurrence among the specific classes that revealed many motifs, that are not reported so far in any NBS-LRR family of available plant genomes (Table 3). These new motifs might have evolved in apple in response to rapidly evolving pathogens and may be responsible in imparting disease resistance. Further studies would be required to ascertain their specific role(s) in apple.

The genomic organization of MdNBS genes shows that the highest percentage of MdNBS genes was present on chromosome 2 (∼15.6%) which is in agreement with previous studies [24], [45]. Clustering of mapped MdNBS genes shows that ∼81% (751 out of 928) of MdNBS genes are present in clusters with average number of genes per cluster approximately five (∼4.7). Similarly, Perazzolli et al. [45] reported 80% (622 out of 778 mapped NBS-LRRs) of MdNBS genes present in clusters, however, with marginally lower average number of genes per cluster (∼4).

We also checked for the possibility of duplication events in NBS-LRRs in apple and other members of Rosaceae family (*Prunus persica* and *Fragaria vesca)*. In *Arabidopsis*, it has been reported that NBS-LRR family follows moderate tandem with low segmental duplication [41]. It was reported that the tandem duplication played a major role in expansion of NBS-LRR families in soybean [43], maize [44] and rice [9], [10]. Among the members of Rosaceae family, *P. persica* has ∼36% tandem and ∼2% segmental duplication, while *F. vesca* has ∼24% tandem and 0.5% segmental duplication (Table 4). This infers that *P. persica* and *F. vesca* also follow same trend of duplication as in case of *Arabidopsis*, with moderate tandem and low segmental duplication events. Intriguingly, MdNBS family shows a high percentage of segmental duplication events (13%) along with tandem duplications (25%). MdNBS family seems to be largest NBS-LRR family in plants, which may be due to higher percentage of segmental duplication events as compared to other plants. In a previous study [24], genome duplication analysis in apple has shown a strong collinearity between chromosome pairs 3 and 11; 5 and 10; 9 and 17; 13 and 16. We also found similar pattern of gene duplication of MdNBS gene family. For instance, the high number of collinear pairs was observed between chromosome 3 and 11; 5 and 10; and 9 and 17.

**Table 4:** Duplication events of NBS-LRRs in Rosaceae family.

Type of Duplication	*Malus domestica (%)*	*Prunus persica (%)*	*Frageria vesca (%)*
**Singleton**	0	1 (0.24)	0
**Dispersed**	311(30.67)	78 (18.93)	96 (50.79)
**Proximal**	317 (31.26)	177(42.96)	47 (24.87)
**Tandem**	254 (25.04)	148 (35.92)	45 (23.81)
**WGD or Segmental**	132 (13.01)	8 (1.94)	1 (0.53)

Of the total 1015 identified MdNBS genes, 83 were found to be specifically expressed in either leaf or shoot tissue using publicly available EST database. These 83 MdNBS genes were taken for phylogenetic analysis along with known NBS genes involved in disease resistance in other plants. On the basis of phylogenetic analyses with known disease resistance genes (Figure S3), 28 MdNBS genes were predicted which might have some role in providing resistance against different pathogens, but primers could be designed only for 26 MdNBS genes as four genes exist in two isoforms. In order to study the expression of these predicted genes in apple tissues infected with different pathogens, the qRT-PCR expression profiling was done.

MdNBS533 and MdNBS223, belong to N class of MdNBS genes, were found to express in all the samples irrespective of pathogen source. A recent study by Su-hua et al. [48], also reported the expression of MdNBS gene (MDP0000137959) in response to exogenous salicylic acid in apple. Thus, up-regulation of N class genes in response to pathogens and defense hormone indicates that MdNBS genes with only NBS domain also have some role in disease resistance. The NBS-LRR genes (MdNBS228, MdNBS231, MdNBS586, MdNBS618 and MdNBS626) exhibited significant up-regulation under different pathogen infection in most of the tissues. Our results are in agreement with findings of Li et al. [49], who reported the higher expression of *PnAG_3_*, a NBS-LRR gene, in peanut fruit tissues of *Aspergillus flavus* resistant variety. Further, over-expression of a pepper NBS-LRR gene, *Bs2* in transgenic tomato conferred increased resistance against bacterial spot disease along with substantial increase in yield [50]. Similarly, higher expression of downy mildew resistance gene *RPP8* in Arabidopsis was reported by Mohr et al. [51] in response to *Hyaloperonospora arabidopsidis* and salicylic acid.

The CC-NBS-LRR, another class of NBS-LRR genes, also plays an important role in disease resistance. Feuillet et al. [52] reported that over expression of a CC-NBS-LRR gene, *Lr10* in wheat leads to increase in resistance against leaf rust. Over-expression of an NBS-LRR gene, *Pi54* in rice was shown to confer resistance against various strains of the fungus *Magnaporthe oryzae* [53]. Similarly, Das et al. [54] observed that transgenic rice plant over expressing *Pi54rh* (CC-NBS-LRR) exhibit broad spectrum resistance against diverse isolates of *M. oryzae.* Gong et al. [55] has shown the higher expression of CC-NBS-LRR gene, TdRGA-7Ba in wheat leaf infected with powdery mildew. Similarly, *Pi36* that encode CC-NBS-LRR was found to confer resistance against rice blast fungus [56]. These studies suggest that MdNBS340, MdNBS502 and MdNBS737 which are up-regulated under all the pathogen infections analyzed in the present study, may have role in imparting disease resistance to plant.

The *RPS4* gene in *Arabidopsis* confers disease resistance against *Pseudomonas syringae* and its TIR domain is hypothesized to transduce signal to downstream component of plant defense signaling pathway [57]. MdTIR-NBS-LRR1 (MDP0000465174) was recently reported to have higher expression in apple on salicylic acid treatment [48]. Members of TNL class were also reported to impart resistance against diverse pathogens in various plants. The *CSRGA23*, a NBS-LRR gene with TIR domain shows higher expression in response to *Pseudoperonospora cubensis* and exogenous application of stress related hormones in downy mildew resistant *Cucumis* [58]. Another study in Pepper [59] reported the high level of expression of TIR-NBS-LRR genes (*CaRGA01*, *CaRGA05*, *CaRGA49*) in leaf, root, stem and seedling in response to defense signaling molecules. Similar to previous findings, higher expression of MdNBS236, MdNBS291 and MdNBS638 was observed in leaf tissue in response to different pathogens. Interestingly, the TIR-NBS-LRR genes of different plants are capable of alternative splicing and generate truncated transcripts. Zhang and Gassmann [60] observed that alternatively spiced (intron deficient) and truncated transcript forms of *RPS4* were required for the partial but sufficient disease resistance in A*rabidopsis.* They also observed that truncated forms work at protein level not as a regulatory RNA to impart the disease resistance. Thus, TIR-NBS genes may be the truncated forms of TIR-NBS-LRR gene family and are essential for disease resistance.

In *Arabidopsis*, expression of four TIR-NBS (TN) genes was found to be higher upon the treatment with different pathogens and other stress signaling molecules. Transgenic *Arabidopsis* plants over-expressing TN genes, AtTN10 and AtTN21 have developed resistance against bacterial pathogens, and also show higher expression in response to salicylic acid [61]. Similarly, exogenous application of salicylic acid was observed to up-regulate the expression of MdTIR-NBS1 (MDP0000386726) in apple [48]. Overexpression of MbR4, a gene belonging to TN class was reported to confer resistance against *Pseudomonas syringae* in transgenic Arabidopsis [62]. In the present study, MdNBS70, a TIR-NBS gene, was found to be significantly up-regulated in leaf tissue infected with different pathogens and insect pest. The higher expression of MdNBS70 under pathogen infection indicates its role in providing resistance to biotic stress.

Up-regulation of most of the MdNBS genes under pathogen infection as analyzed by qRT-PCR suggests that the sequence similarity-based targeted gene identification approach has high degree of accuracy. Interestingly, MdNBS231 and MdNBS638 identified in the present analysis, not reported previously [24], [45], have shown their higher expression in response to pathogen attack, thus further validate the methodology used to predict NBS-LRRs in apple. Moreover, the tissue specific expression profiling will help to identify the decisive role of these genes in individual tissue in conferring the resistance against pathogens. Importantly, pathogen-responsive NBS-LRR genes identified in present study may be used as candidate genes for engineering pathogen resistance in apple and also in other related species.

## Supporting Information

Figure S1 The flowchart depicts the methodology used for selecting MdNBS genes for qRT-PCR analysis.
(TIF)

Figure S2 Representative images of various disease incidences. Symptoms of Alternaria (A); Powdery Mildew (B); Apple Mosaic Virus (C); Sap sucking insect (D) infected apple leaf.
(TIFF)

Figure S3 Phylogenetic tree for 83 MdNBS genes (specifically expressed in shoot and leaves tissues) with well-known plant disease resistance genes. The MdNBS genes selected for qRT-PCR analysis were shown in bold letters.
(TIF)

Table S1 List of primers used for qRT-PCR analysis of selected MdNBS genes.
(DOC)

Table S2 Identified apple NBS-LRRs are listed along with MDP identity in the phytozome, MdNBS identity, chromosome number, location (starting and ending position), type of NBS-LRR class, classification as regular/non-regular NBS-LRRs and duplication pattern of NBS-LRRs.
(XLS)

Table S3 List of apple NBS-LRRs having RPW8 domain classified as CC_R-_NBS-LRR.
(DOC)

Table S4 List of gene bank accession number (NCBI) of known resistance genes used for phylogenetic analysis.
(XLS)

Table S5 List of MdNBSs analyzed for digital expression in tissue types shoot, root, leaves, xylem and phloem.
(XLS)

Table S6 List of comparison of individual NBS-LRRs found by previous two studies [24], [45] and present study.
(XLS)
