# Supplementary figures and images for: Genome-Wide Identification and Expression Analysis of NBS-Encoding Genes in Malus x domestica and Expansion of NBS Genes Family in Rosaceae
Source: PLoS One. 2014 Sep 18;9(9):e107987. doi: 10.1371/journal.pone.0107987 (PMC4169499; doi:10.1371/journal.pone.0107987)

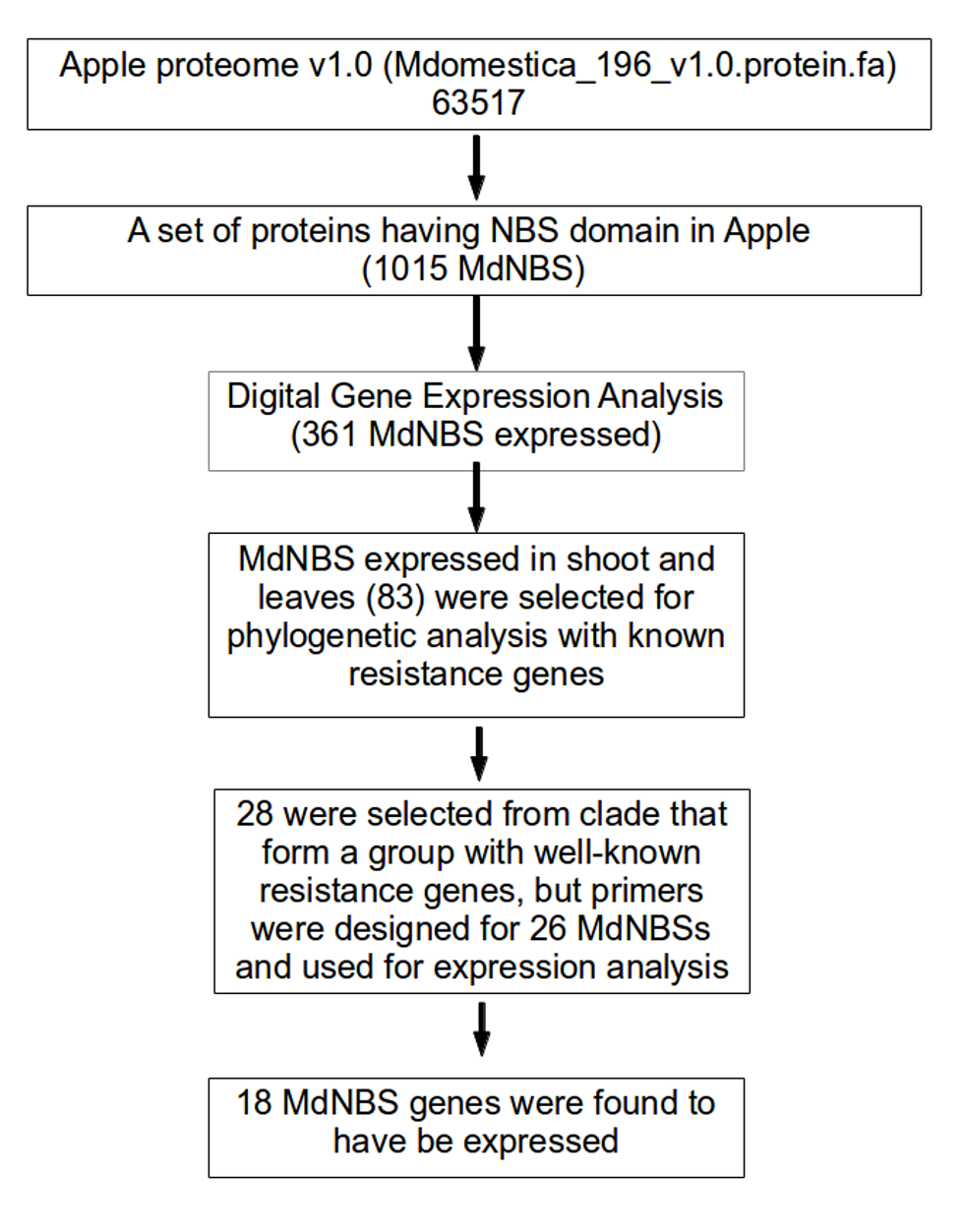

Supplement: Figure S1 — The flowchart depicts the methodology used for selecting MdNBS genes for qRT-PCR analysis. (TIF) [file pone.0107987.s001.tif]

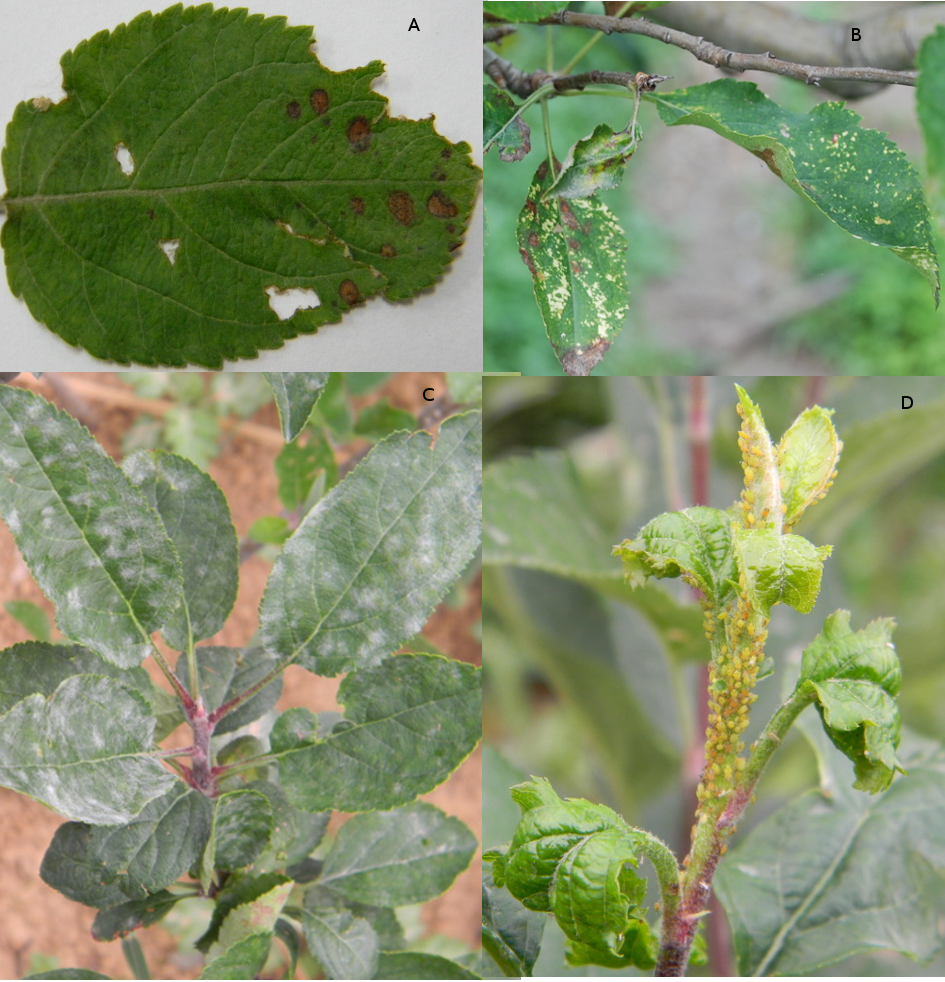

Supplement: Figure S2 — Representative images of various disease incidences. Symptoms of Alternaria (A); Powdery Mildew (B); Apple Mosaic Virus (C); Sap sucking insect (D) infected apple leaf. (TIFF) [file pone.0107987.s002.tiff]
