# Supplementary material for: Genome-Wide Identification and Expression Analysis of NBS-Encoding Genes in Malus x domestica and Expansion of NBS Genes Family in Rosaceae
Source: PLoS One. 2014 Sep 18;9(9):e107987. doi: 10.1371/journal.pone.0107987 (PMC4169499; doi:10.1371/journal.pone.0107987)
Supplement: Table S1 — List of primers used for qRT-PCR analysis of selected MdNBS genes. (DOC) [file pone.0107987.s004.doc]

Table S1: List of primers used for qRT-PCR analysis of selected MdNBS genes.

| **Accession No.** | **Prime name** | **Primer sequence** | **Amplicon length (bp)** |
| --- | --- | --- | --- |
| MDP0000758643 | MdNBS-70 F | 5’-CCTTGCTGAAACTGTATTTCACAAA-3’ | 107 |
|  | MdNBS-70 R | 5’-CCAAGTGATCTAGTCCATCTGTT-3’ |  |
| MDP0000643262 | MdNBS-184 F | 5’-ACCTTGAGAATCTACAGATTCAAG-3’ | 147 |
|  | MdNBS-184 R | 5’-TCGGTGATCTCAGCAACTTCAT-3’ |  |
| MDP0000296620 | MdNBS-223 F | 5’-CTCATGCTCAGGTATGGATCAAA-3’ | 125 |
|  | MdNBS-223 R | 5’-CCTTTCCCTTGTAGCTGAATCTA-3’ |  |
| MDP0000300921 | MdNBS-228 F | 5’-TAGAGAAGCTGCCAGAATACCAT-3’ | 112 |
|  | MdNBS-228 R | 5’-AAGTTCGGCAGTTTTTCCAGTGT-3’ |  |
| MDP0000291857 | MdNBS-231 F | 5’-TGGCCGAGAAATAGTAGATAAAG-3’ | 109 |
|  | MdNBS-231 R | 5’-CAGATCTAGAACTCTCAACAACT-3’ |  |
| MDP0000218361 | MdNBS-236 F | 5’-CTTGGTCTGGTCCAGATGATAT-3’ | 119 |
|  | MdNBS-236 R | 5’-GCCATCGGCTCCATAATAAACAT-3’ |  |
| MDP0000131151 | MdNBS-274 F | 5’-AGGTTTTGGTACATCCATTTGCTT-3’ | 102 |
|  | MdNBS-274 R | 5’-TTTCTCGGCAAGCTCTGCTTT-3’ |  |
| MDP0000153244 | MdNBS-275 F | 5’-CAAGAAAAGAAAGAGCGAAGTCTT-3’ | 103 |
|  | MdNBS-275 R | 5’-GCCTAATGATTCTCTCTTCCTCA-3’ |  |
| MDP0000811127 | MdNBS-276 F | 5’-ACATTTCTTCCCCTCCTCAGTT-3’ | 99 |
|  | MdNBS-276 R | 5’-GTCTGGCCAAACTGTTAAGTGA-3’ |  |
| MDP0000811129 | MdNBS-282 F | 5’-GAGCATCTGACAGAGTTGAAAGT-3’ | 106 |
|  | MdNBS-282 R | 5’-GGATGTGCAAGACTTTGGAATTAT-3’ |  |
| MDP0000540495 | MdNBS-291 F | 5’-CCAACGATTAATGGCTGCTACTT-3’ | 119 |
|  | MdNBS-291 R | 5’-CTTTGTAGAGATGGCTGACGAA-3’ |  |
| MDP0000392360 | MdNBS-292 F | 5’-TTCATCTTCTTCTGGCCTTGGTT-3’ | 106 |
|  | MdNBS-292 R | 5’-CTGACGCAGAGCGTTGTAGA-3’ |  |
| MDP0000232833 | MdNBS-302 F | 5’-GATTATGTTCGGTTTAAGAGGACTT-3’ | 105 |
|  | MdNBS-302 R | 5’-TTCGCTCTGATCAAGCTCATTCT-3’ |  |
| MDP0000241462 | MdNBS-309 F | 5’-AGGATGGGGTGGTCACCAAA-3’ | 98 |
|  | MdNBS-309 R | 5’-CCAATTCAGCAAGACTCAACATC-3’ |  |
| MDP0000243301 | MdNBS-310 F | 5’-GTTGTAAAATGCACGATCTCATGA-3’ | 112 |
|  | MdNBS-310 R | 5’-GATACATGACGAACCTTTTCATGA-3’ |  |
| MDP0000772634 | MdNBS-340 F | 5’-TCATCCAAAGACTCCCAGATCTT-3’ | 120 |
|  | MdNBS-340 R | 5’-ACTGGCAAATATTGGAGCTTATCA-3’ |  |
| MDP0000321938 | MdNBS-385 F | 5’-ATCTCATTGACCGCATGCAAC-3’ | 137 |
|  | MdNBS-385 R | 5’-CTTATTTCCACGGAGAATGCAAC-3’ |  |
| MDP0000285649 | MdNBS-502 F | 5’-CATCGTTTACTTGCAGTCAGGAT-3’ | 120 |
|  | MdNBS-502 R | 5’-GTGCTTCCAGTTCAATTACCTGA-3’ |  |
| MDP0000752582 | MdNBS-533 F | 5’-TCCGAAAAGTAGCAAAAGAGGTAT-3’ | 115 |
|  | MdNBS-533 R | 5’-CATTCCTTCTTAGCCTTCACCTT-3’ |  |
| MDP0000197733 | MdNBS-586 F | 5’-CTTAATCAAGTTGGGTCTGAGATT-3’ | 102 |
|  | MdNBS-586 R | 5’-CTGGTGATCTCTTACAACACTCA-3’ |  |
| MDP0000249728 | MdNBS-618 F | 5’-CTTGGGATTCGCTCTATCTGTT-3’ | 97 |
|  | MdNBS-618 R | 5’-CTCAAACTGCATACCTGGTTGAT-3’ |  |
| MDP0000300756 | MdNBS-626 F | 5’-ATGATCTTGCAGAACGGCTGAAA-3’ | 117 |
|  | MdNBS-626 R | 5’-TCTTGGCTTGAGCATATTCTTTCA-3’ |  |
| MDP0000416021 | MdNBS-638 F | 5’-CAACACAACTTGTCAATGGAAGTA-3’ | 114 |
|  | MdNBS-638 R | 5’-GTTTTGACGTTTTTGTCCTGCAAT-3’ |  |
| MDP0000248430 | MdNBS-640 F | 5’-CAAGACAACCATTGCTAGACTAG-3’ | 107 |
|  | MdNBS-640 R | 5’-CATGCATACTTACATTGCTCTCTA-3’ |  |
| MDP0000308012 | MdNBS-737 F | 5’-TACTTCTCACTCCCCCTCTAT-3’ | 116 |
|  | MdNBS-737 R | 5’-AGACACCACGACATCGAAGTA-3’ |  |
| MDP0000201899 | MdNBS-756 F | 5’-GCTGGAGGACTAAAAGCATGTA-3’ | 130 |
|  | MdNBS-756 R | 5’-AGCGAAGCTCTGACATTCGTA-3’ |  |
| MDP0000290336 | MdRPL2 F | 5’-GCCACCCCTTCCGTTACAA-3’ | 102 |
|  | MdRPL2 R | 5’-CCACCAAGTTGGCCTTCTTC-3’ |  |
| rRNA Inter-spacer region | ITS1-F | 5’-TCCGTAGGTGAACCTGCGG-3’ | 570 |
|  | ITS4-R | 5’-TCCTCCGCTTATTGATATGC-3’ |  |
| ApMV Coat protein | CpF | 5’-CTCAAGCGAACCCGAATAAGGGTAAGAA-3’ | 547 |
|  | CpR | 5’-TCGTCGATAAGTAGAACATTCGTCGGTATTGTC-3’ |  |
