# Supplementary material for: Genome-Wide Identification and Expression Analysis of NBS-Encoding Genes in Malus x domestica and Expansion of NBS Genes Family in Rosaceae
Source: PLoS One. 2014 Sep 18;9(9):e107987. doi: 10.1371/journal.pone.0107987 (PMC4169499; doi:10.1371/journal.pone.0107987)
Supplement: Table S3 — List of apple NBS-LRRs having RPW8 domain classified as CCR-NBS-LRR. (DOC) [file pone.0107987.s006.doc]

Table S3**:** NBS-LRR containing RPW8 domain present in apple.

| **MdNBS genes** | **Class of MdNBS genes** |
| --- | --- |
| MdNBS99 | NBS-LRR |
| MdNBS100 | CC_R_-NBS-LRR |
| MdNBS101 | CC_R_-NBS-LRR |
| MdNBS102 | CC_R_-NBS-LRR |
| MdNBS103 | CC_R_-NBS-LRR |
| MdNBS107 | NBS-LRR |
| MdNBS128 | NBS |
| MdNBS134 | NBS-LRR |
| MdNBS137 | NBS-LRR |
| MdNBS147 | NBS-LRR |
| MdNBS197 | NBS-LRR |
| MdNBS209 | NBS-LRR |
| MdNBS274 | CC_R_-NBS |
| MdNBS275 | CC_R_-NBS |
| MdNBS279 | NBS |
| MdNBS366 | CC_R_-NBS-LRR |
| MdNBS380 | NBS |
| MdNBS394 | NBS-LRR |
| MdNBS657 | CC_R_-NBS |
| MdNBS659 | CC_R_-NBS-LRR |
| MdNBS661 | CC_R_-NBS |
| MdNBS663 | CC_R_-NBS |
| MdNBS674 | NBS |
| MdNBS683 | NBS-LRR |
| MdNBS819 | CC_R_-NBS-LRR |
| MdNBS822 | NBS |
| MdNBS985 | NBS |

R  Coiled coil domain with RPW8 domain
